# Hypolipidemic effect of *Fragarianilgerrensis* Schlecht. medicine compound on hyperlipidemic rats

**DOI:** 10.1186/s12944-018-0868-4

**Published:** 2018-09-19

**Authors:** Liangcai Gao, Zejie Lin, Yilian Liu, Xinyi Wang, Linlin Wan, Liuliu Zhang, Xinnan Liu

**Affiliations:** School of Life Science, East China Normal University, Shanghai, 200241 China

**Keywords:** *Fragarianilgerrensis* Schlecht., *Centella asiatica (L.)* urban., Hyperlipidemia, Antioxidation, Histopathology

## Abstract

**Background:**

*Fragarianilgerrensis* Schlecht. medicine compound (FN-MC) is a kind of Chinese herbs’ compound consisted of *Fragarianilgerrensis* Schlecht. and *Centella asiatica (L.)* Urban. The study was to investigate the hypolipidemia effect of FN-MC in a hypolipidemic rat model.

**Methods:**

Male SD rats were randomly divided into five groups: normal-fat diet (NFD) group, high-fat diet (HFD) group, FN-MC (2 g/Kg) group, FN-MC (4 g/Kg) group and simvastatin (PDC) group. After FN-MC treatment, body weight, food intake, serum and hepatic biochemistry parameters of rats were measured and the pathological changes of liver and its cells were observed by optical microscope and transmission electron microscopy.

**Results:**

The results showed that FN-MC significantly decreased the levels of serum triglyceride (TG), total cholesterol (TC), low-density lipoprotein (LDL-C), apolipoprotein B (ApoB) and hepatic malondialdehyde (MDA), while increased serum high-density lipoprotein (HDL-C), apolipoprotein A1 (ApoA1) and hepatic Superoxide Dismutase (SOD). FN-MC also improved the structure of liver and decreased the lipid drops in the cytoplasm significantly. In addition, FN-MC significantly decreased the weight gain and had no significant effects on food intake.

**Conclusions:**

The study suggested that FN-MC exhibited strong ability to improve the dyslipidemia and prevent hepatic fatty deposition in rats fed with high-fat diet. Meanwhile, FN-MC exerted anti-obesity and antioxidant properties.

**Highlights:**

- Fragarianilgerrensis Schlecht. medicine compound possesses a hypolipidemic effect on hyperlipidemic rat model
- Fragarianilgerrensis Schlecht. medicine compound administration improves the antioxidant capacity of rats
- Fragarianilgerrensis Schlecht. medicine compound prevents hepatic fatty deposition

## Background

Hyperlipidemia is a kind of metabolic disorder disease which involves an abnormally high level of blood lipids and lipoproteins. It is a major cause of arteriosclerosis, cerebral stroke, coronary heart disease, myocardial infarction and renal failure in Chinese people [1–3].

At present, the main treatment of hyperlipidemia is exercise, smoking cessation, dietary therapy and medication. Statins, nicotinic acids and bile acid sequestrants are the most commonly used medicine by far which can reduce lipids and lipoproteins in the blood. It has been shown that those drugs are beneficial in patients with preventing many kinds of cardiovascular disease [4]. Although they are effective in modulating hyperlipidemia in both preclinical and clinical studies, their toxicity of liver and kidney can’t be ignored, so a lot of researchers are working on finding more effective drugs to put into treatment [5–7]. In recent years, Chinese medicine has received great attention of many scholars for its stabilizing effect and few side-effects [6, 7].

*Fragarianilgerrensis* Schlecht. is a plant which belongs to Rosaceae, Fragaria. It grows at grasslands of mountain slope or forests on river bank which are at an altitude of 700-3000 m and it mainly distributes in Yunnan, Guangxi of China. *Fragarianilgerrensis* Schlecht. has perfect effect of heat-clearing and detoxifying and it can activate blood circulation to dissipate blood stasis. People in Yunnan, China usually make it into mixture with *Centella asiatica (L.)* Urban [8, 9] and use the mixture as a folk remedy to treat cardiovascular diseases.

In our experiment, we fed the rats with high-fat diet to establish hyperlipidemic rat model. Then we divided the rats into NFD group, HFD group, FN-MC (2 g/Kg) group, FN-MC (4 g/Kg) group and PDC group. After the drug treatment, total cholesterol(TC), triglyceride (TG), low density lipoprotein (LDL-C), high density lipoprotein (HDL-C), apolipoprotein A1 (ApoA1) and apolipoprotein B (ApoB) in serum were measured to evaluate the hypolipidemic effect of FN-MC [10–12]. Meanwhile, we used optical microscope and electron microscopy to observe the changes of morphology and ultrastructure of liver tissue. In addition, we measured the activity of superoxide dismutase (SOD) and the content of malondialdehyde (MDA) in the liver homogenate to study the changes of rats’ antioxidant capacity [13–15].

## Methods

### Drug preparation

The whole plants of *Fragarianilgerrensis* Schlecht. and *Centella asiatica (L.)* Urban (including roots, stems and leaves) were cut up after drying. Each herbal medicine weighed 12 g, and leaching by 500 ml of 75% ethanol at room temperature for 3 h. Cooked and concentrated the compound under vacuum in a rotary evaporator at 40 ± 5 °C for 1.5 h and then filtrate to obtain the extract to make the compound whose crude drug concentration was 0.4 g • ml^− 1^ [16].

The dose of simvastatin for human is 20 mg per day. The dose for rat was calculated by the human equivalent dose (HED) on the basis of body surface area: assuming a human weight of 60 kg, the HED for 20 (mg)/60 (kg) = 1/3 mg/kg [16]; rat dose converted by body surface area was 2 mg/kg; the experimental effective dose was twice as high as the clinical dose, so it was 4 mg/kg. Each simvastatin capsule was dissolved in 50 ml of water to form a simvastatin solution at a concentration of 0.4 mg / ml.

### Experimental animals

A total of 30 male Sprague-Dawley rats (180 ± 20 g body weight) were obtained from Shanghai Slac Laboratory Animal Co., Ltd.(Shanghai, China). The rats were housed at 22 ± 2 °C with free access to food and water, under a 12:12 h light/dark cycle (lights on at 08:00 h). All experimental methods were approved by the Institutional Review Boards of East China Normal University, and they were performed in accordance with relative guidelines and regulations.

### Animal grouping

After acclimation for two weeks, the rats were randomly divided into two groups: normal-fat diet (NFD) group (*n* = 6) and high-fat diet (HFD) group (*n* = 24). Rats of HFD group were fed with high-fat fodder of 60% calorie for three weeks to establish the hyperlipidemic model. After 3 weeks, the hyperlipidemic rats were took the blood sample from tail vein to measure the lipids and subdivided into 4 groups (n = 6 in each group). (1) HFD group: HFD with 10 ml/kg/day distilled water; (2) FN-MC (2 g/kg) group: HFD with 10 ml/kg/day 0.2 g·ml^− 1^ FN-MC; (3) FN-MC (4 g/kg) group: HFD with 10 ml/kg/day 0.4 g·ml^− 1^ FN-MC; (4) PDC group: HFD treated with 10 ml/kg/day 0.4 g·ml^− 1^ simvastatin solution. Rats of NFD group were given the same volume of distilled water equivalent to body weight. Distilled water and drugs were given by gavage once a day for 3 weeks.

### Sample collection

During the experimental period, body weight was recorded once a week and the food intake of each group was recorded daily. After 3 weeks, blood from each rat was collected from caudal vein after an over-night fasting. The collected whole blood was kept in refrigerator in 4 °C for 15 min, then serum was obtained after the blood was centrifuged (3000 rpm for 15 min) and stored at − 80 °C until analysis. The rats were sacrificed by cervical dislocation and tissues were harvested and stored at − 80 °C until use.

### Serum and hepatic biochemical parameters analysis

The serum total cholesterol (TC), triglycerides (TG), high-density lipoprotein cholesterol (HDL-C) and low-density lipoprotein cholesterol (LDL-C) were measured using commercial kits (Nanjing Jiancheng Bioengineering Institute, Nanjing, China) according to the manufactures’ instructions. The serum apolipoprotein A1 (ApoA1) and apolipoprotein B (ApoB) were measured by commercial kits (Westang Bio-Tech, Shanghai, China) according to the manufacturer’s protocols. A part of liver was homogenized in ice cold PBS. The homogenate was centrifuged and the supernatant was taken to measure the activity of SOD (xanthine oxidase method) and the content of MDA (TBA method, thiobarbituric acid method), by using commercial kits (Nanjing Jiancheng Bioengineering Institute, Nanjing, China) according to the manufactures’ instructions.

### Histological analysis

Liver tissues were removed quickly from rats, fixed in 4% paraformaldehyde solution, and then embedded in paraffin. Sections were obtained and later stained with hematoxylin and eosin (H&E) for the histological examination under microscope.

### Ultrastructural analysis

Liver tissues were isolated from rats, fixed with 2.5% glutaraldehyde solution and 1% osmium acid solution, then dehydrate with a different concentration gradient of ethanol solution and embedded with epoxy resin. The samples were treated with ultrathin microtome and then stained with uranyl citrate-lead citrate. Finally, the ultrastructure of hepatocytes was observed and photographed with transmission electron microscope. Total 36 planes (6 planes per group) were used to determine the surface area of lipid droplets. The surface area of lipid droplets was measured by using Image J software (National Institute of Mental Health, USA).

### Statistical analysis

Values are presented as the mean ± S.E.M. Significant differences among the results of five groups were analyzed by GraphPad Prism 7.0 software using one-way ANOVA followed by Dunnett’s posttest. *P* < 0.05 was considered to be statistically significant.

## Results

### Effect of FN-MC on body weight, weight gain and food intake of rats

**Fig. 1:**
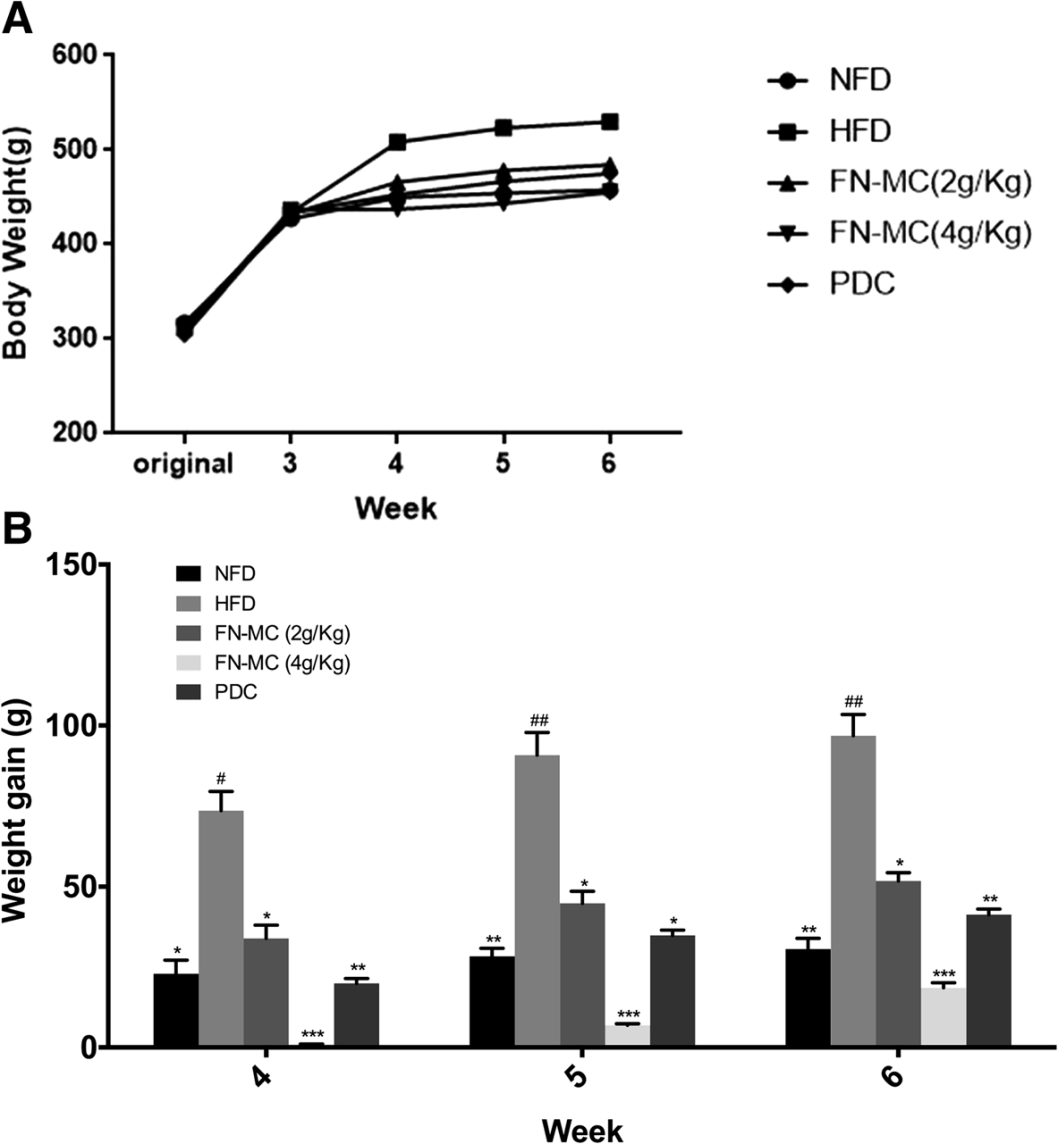
Effect of FN-MC on body weight and weight gain of rats. In the induction phase, rats were separated into the NFD group (n = 6) and HFD group (*n* = 24). After 3 weeks, the 24 rats were randomly assigned to four groups (six rats/group) for solvent, FN-MC (2 g/Kg), FN-MC (4 g/Kg) or simvastatin solution (10 mg/kg/day) administration. *P < 0.05, **P < 0.01, ***P < 0.001 significant differences compared to HFD group; ^#^*P* < 0.05, ^##^*P* < 0.01, ^###^*P* < 0.001 significant differences compared to NFD group

The body weight and the weight gain of rats are shown in Table 1 and Fig. 1. There were no differences in initial body weight among 5 groups in the experiment (*P* > 0.05), indicating that the grouping was reasonable. During the gavage period, the body weight of the rats increased with time, and the weight gain of the rats in HFD group was significantly higher than that of the other four groups. The increment of the rats in FN-MC group (4 g/Kg) was much smaller than that in HFD group and FN-MC (2 g/Kg) also had some effect on body weight. After 1 week of gavage, the body weights of the PDC group and FN-MC group were significantly different from that of HFD group (*P* < 0.05). Gavage for the second and the third week, compared with HFD group, the body weight of FN-MC (4 g/Kg) group had a significant difference (*P* < 0.01). After 4 weeks, although rats treated with FN-MC (2 g/Kg) and FN-MC (4 g/Kg) were fed the high-fat diet, they had a reduced weight gain compared with HFD group (*P* < 0.05, *P* < 0.001, respectively). Meanwhile, the weight gain of PDC group after 4 weeks was also decreased compared with HFD group (*P* < 0.05). After 6 weeks, the total weight gain of FN-MC (4 g/Kg) group was much less than PDC group. However, food intake was no difference between the groups (Table 1). The results suggested that FN-MC reduced the weight gain of mice and had no significant effects on food intake.

**Table 1:** Effect of FN-MC on body mass and food intake of rats(*n* = 6)

Group	Original	Week-3	Week-4	Week-5	Week-6	Food intake (g/d)
NFD	317.48 ± 20.25	427.24 ± 40.36	449.92 ± 13.15*	454.45 ± 11.81**	457.80 ± 13.99**	23.43 ± 3.62
HFD	313.67 ± 16.15	433.44 ± 26.80	508.62 ± 35.15	523.35 ± 31.78^#^	530.25 ± 32.00^#^	21.22 ± 3.39
FN-MC (2 g/kg)	312.43 ± 14.69	432.57 ± 34.47	466.3 ± 19.13*	478.51 ± 19.64*	484.39 ± 23.57*	21.85 ± 4.15
FN-MC (4 g/kg)	309.28 ± 19.77	436.96 ± 26.93	437.4 ± 18.21*	443.37 ± 16.35**	455.52 ± 18.41**	24.69 ± 4.21
PDC	304.41 ± 24.96	433.7 ± 32.71	452.8 ± 14.37*	466.97 ± 16.15*	475.00 ± 17.47*	23.51 ± 4.96

The results were represented as mean ± SEM in each group (n = 6 rats/group). *P < 0.05, **P < 0.01 significant differences compared to HFD group; ^#^*P* < 0.05, ^##^*P* < 0.01 significant differences compared to NFD group

### Effect of FN-MC on serum lipid profiles of rats

**Fig. 2:**
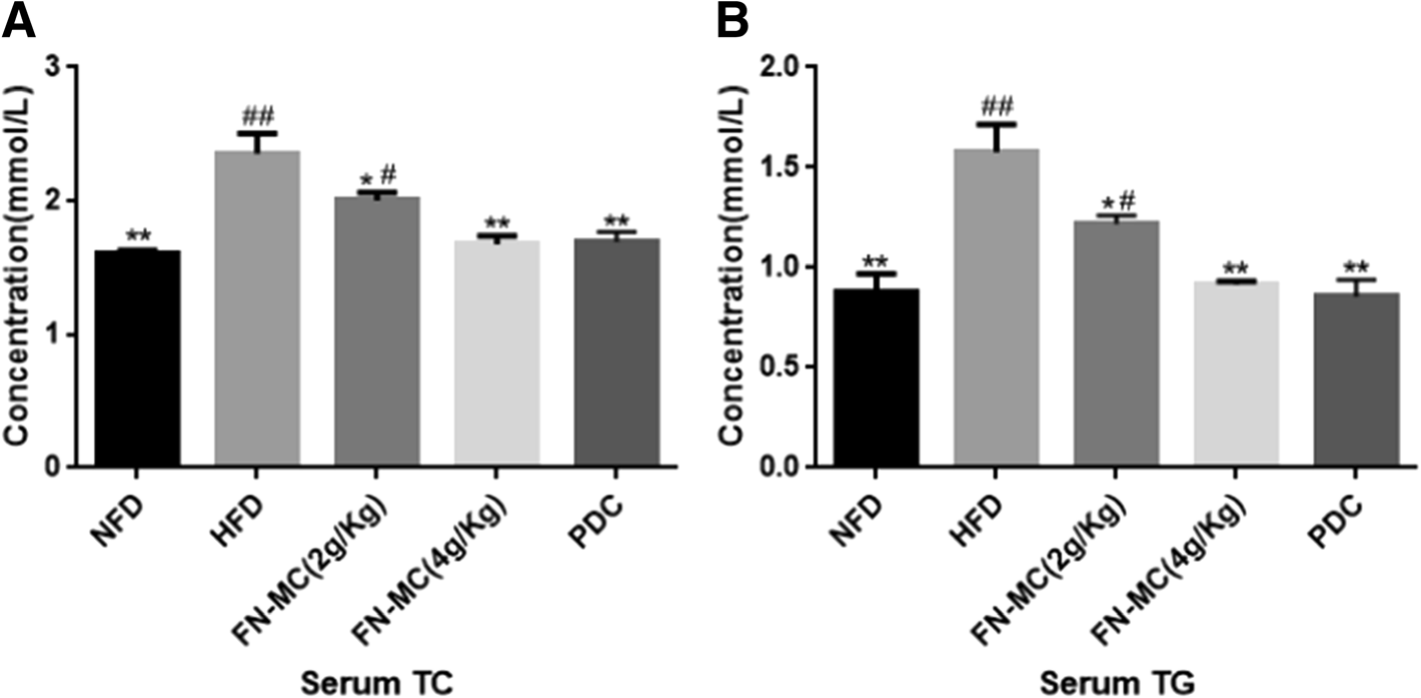
Effect of three-week FN-MC treatment on serum lipid profiles (TC and TG) of rats. *P < 0.05, **P < 0.01 significant differences compared to HFD group; ^#^*P* < 0.05, ^##^*P* < 0.01 significant differences compared to NFD group. TC: total cholesterol; TG: triglyceride

Effects of FN-MC on serum lipid profiles in the experimental rats are shown in Fig. 2. After 3 weeks, the levels of TC and TG in the HFD group were significantly higher than those in NFD group (*P* < 0.01). But after treatment with 2 g/Kg and 4 g/Kg of FN-MC for 3 weeks, compared with HFD group, the levels of TC were significantly decreased by 14.86% (*P* < 0.05) and 28.87% (*P* < 0.01), and the levels of TG were significantly decreased by 22.78% (*P* < 0.05) and 34.86% (*P* < 0.01). The above results indicated that FN-MC had obvious hypolipidemic effect on hyperlipidemia rats and the 4 g/Kg of FN-MC had more capacity to lower the serum lipid profiles.

### Effect of FN-MC on serum lipoprotein profiles of rats

**Fig. 3:**
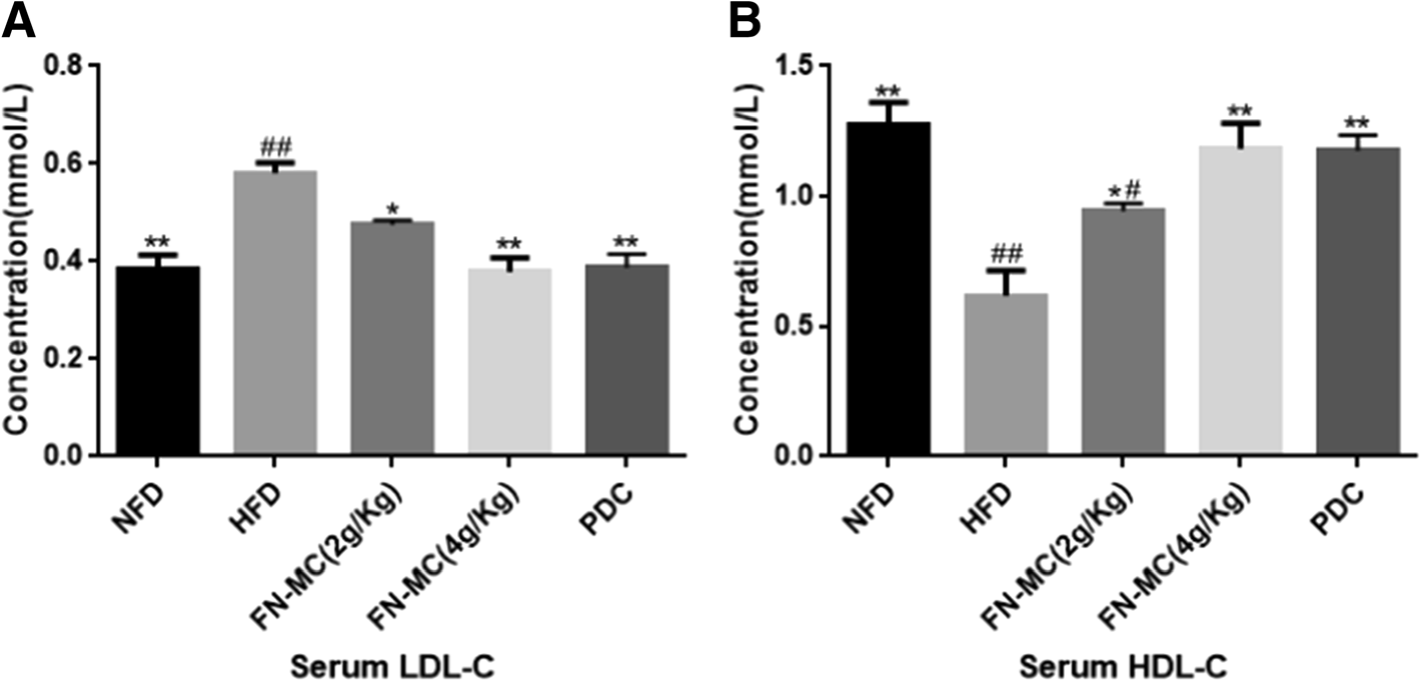
Effect of three-week FN-MC treatment on serum lipoprotein profiles (LDL-C and HDL-C) of rats. *P < 0.05, **P < 0.01 significant differences compared to HFD; ^#^*P* < 0.05, ^##^*P* < 0.01 significant differences compared to NFD. LDL-C: low-density lipoprotein cholesterol; HDL-C: high-density lipoprotein cholesterol

Effects of FN-MC on serum lipid profiles in the experimental rats are shown in Fig. 3. After 3 weeks, the levels of LDL-C in the HFD group were significantly higher than those in NFD group (*P* < 0.01), and the level of HDL-C was significantly decreased (*P* < 0.01). After treatment with FN-MC (2 g/Kg) for 3 weeks, compared with HFD group, the levels of LDL-C were significantly decreased by 18.22% (*P* < 0.05) and the level of HDL-C was significantly increased by 52.80% (*P* < 0.05). But after treatment with FN-MC (4 g/Kg) for 3 weeks, the levels of LDL-C were significantly decreased by 32.70% (*P* < 0.01) and the level of HDL-C was significantly increased by 91.74% (*P* < 0.01), indicating that 4 g/Kg of FN-MC had more obvious hypolipidemic effect on hyperlipidemia rats.

### Effect of FN-MC on serum ApoA1 and ApoB levels of rats

As shown in Table 2, compared with NFD group, the level of ApoB in HFD group was significantly higher (*P* < 0.01) and the level of ApoA1 in HFD group was significantly lower (*P* < 0.05). Meanwhile, the ratio of ApoA1/ApoB in HFD group was significantly lower than NFD group (*P* < 0.001). Compared with HFD group, FN-MC (2 g/Kg) group and FN-MC (4 g/Kg) group showed the decreased ApoB levels and increased ApoA1 levels (*P* < 0.05). The ratios of ApoA1/ApoB in FN-MC (2 g/Kg) group and FN-MC (4 g/Kg) group were significantly higher(*P* < 0.001). After treatment with PDC, the ratio of ApoA1/ApoB was increased compared with HFD group (*P* < 0.001). The results indicated that FN-MC had regulatory effects on the apolipoprotein level of high-fat diet mice.

**Table 2:** Effect of FN-MC on serum ApoA1 and ApoB levels of rats(*n* = 6)

Group	ApoA1(mmol/L)	ApoB(mmol/L)	ApoA1/ ApoB
NFD	0.55 ± 0.08*	0.34 ± 0.03**	1.62 ± 0.04***
HFD	0.31 ± 0.04^#^	0.61 ± 0.06^##^	0.51 ± 0.06^###^
FN-MC (2 g/kg)	0.43 ± 0.03*	0.46 ± 0.03*	0.93 ± 0.03***
FN-MC (4 g/kg)	0.48 ± 0.03**	0.41 ± 0.05*	1.17 ± 0.04***
PDC	0.49 ± 0.04**	0.41 ± 0.03*	1.20 ± 0.04***

The results were represented as mean ± SEM in each group (n = 6 rats/group). *P < 0.05, **P < 0.01, ***P < 0.001 significant differences compared to HFD group; ^#^*P* < 0.05, ^##^*P* < 0.01, ^###^*P* < 0.001 significant differences compared to NFD group

### Effect of FN-MC on liver antioxidant capacity of rats

**Fig. 4:**
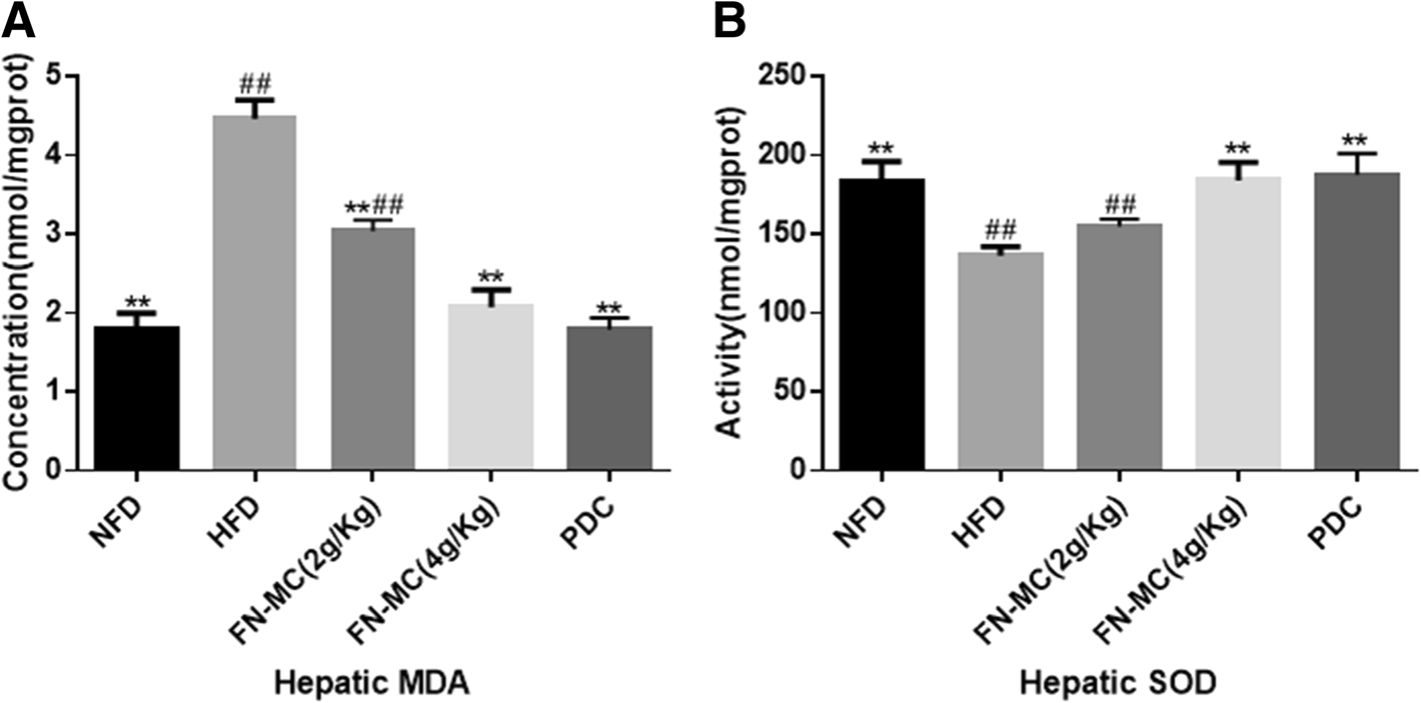
Effect of three-week FN-MC treatment on liver antioxidant capacity of rats. *P < 0.05, **P < 0.01 significant differences compared to HFD; ^#^*P* < 0.05, ^##^*P* < 0.01 significant differences compared to NFD. MDA: Malondialdehyde; SOD: Superoxide dismutase

As shown in Fig. 4, compared with NFD group, the content of MDA in the liver of HFD group was significantly increased (*P* < 0.01), and the activity of antioxidant enzyme SOD was significantly decreased. Compared with HFD group, the content of MDA in the liver of FN-MC (2 g/Kg and 4 g/Kg) group was significantly decreased by 31.90% (*P* < 0.01) and 53.67% (*P* < 0.01) and the activity of SOD was significantly increased by 13.58% (*P* < 0.01) and 33.36% (*P* < 0.01), which was similar to that of PDC group. The results indicated that FN-MC had a significant improvement on the antioxidant ability of liver tissue of hyperlipidemic rats.

### Effects of FN-MC on morphology and ultrastructure of liver tissue in rats

**Fig. 5:**
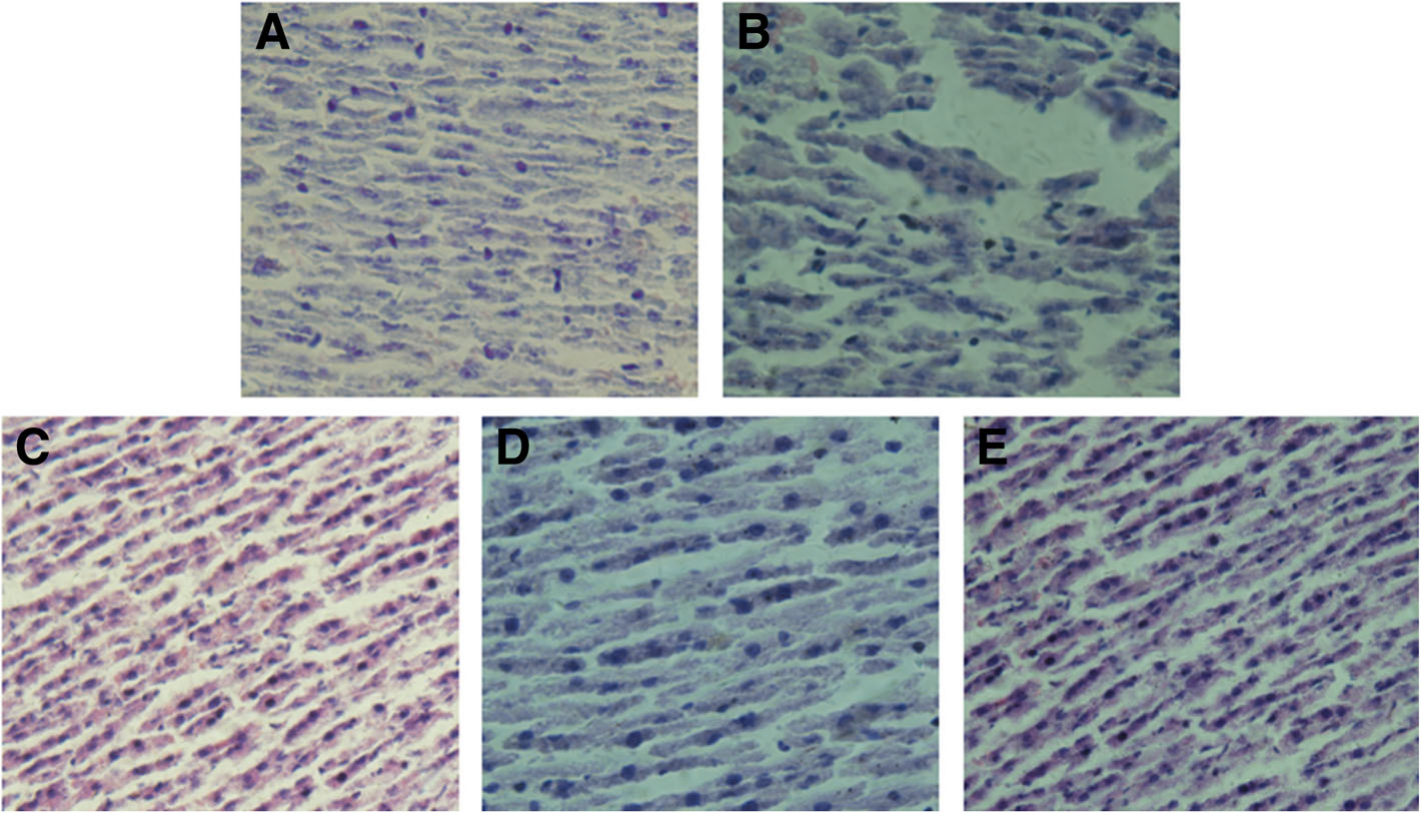
Histopathological sections of liver tissue in rats. Original magnification 400× and HE staining. **a** NFD Group; **b** HFD Group; **c** FN-MC (2 g/Kg) Group; **d** FN-MC (4 g/Kg) Group; **e** PDC Group)

**Fig. 6:**
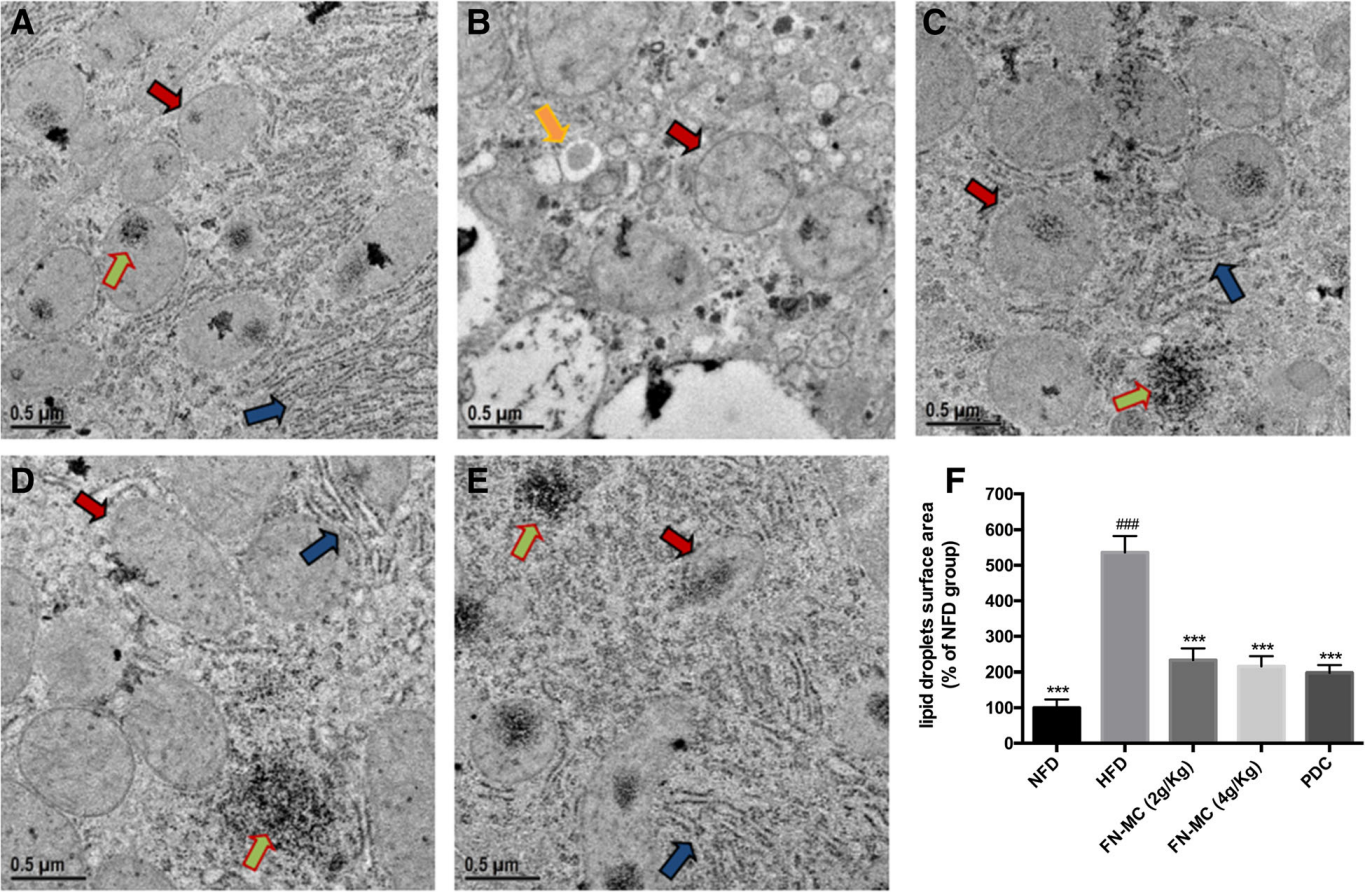
Effects of FN-MC on ultrastructure of liver tissue in rats. Represents mitochondria, represents endoplasmic reticulum (ER), represents glycogen granules, represents lipid droplets **a** NFD Group; **b** HFD Group; **c** FN-MC (2 g/Kg) Group; **d** FN-MC (4 g/Kg) Group; **e** PDC Group; **f** Quantitative analysis of lipid droplets surface area: Mean surface area for lipid droplets was measured using Image J software. Data are mean ± SEM (n = 6 rats/group).) *P < 0.05, **P < 0.01, ***P < 0.001 significant differences compared to HFD group; ^#^*P* < 0.05, ^##^*P* < 0.01, ^###^*P* < 0.001 significant differences compared to NFD group

Effects of FN-MC on histopathological sections of liver tissue, ultrastructure of liver tissue and quantitative analysis of lipid droplets surface area are shown in Fig. 5 and 6. In the liver section of the NFD group, the structure of hepatic lobular was clear and intact. Hepatocytes were arranged in the hepatic cords (Fig. 5a). There were few lipid droplets in the hepatocytes, and a large number of mitochondria and endoplasmic reticulum (ER) were found in the cytoplasm. The mitochondria did not show swelling, and the RER was clear and dense (Fig. 6a and f. However, the liver tissues of HFD group were disordered and the liver cells have necrosis partly (Fig. 5b). There were a large number of lipid droplets but a small number of mitochondria in the cytoplasm. The mitochondria were swelling and few RER or glycogen granules were found in the liver cells (Fig. 6b, and f). After the treatment with FN-MC, the liver tissues returned to normal status (Fig. 5c, and d). In the cytoplasm, the lipid droplets reduced significantly and the mitochondria was rich but still swelling. At the same time, the number of RER and glycogen granules in the cytoplasm increased clearly which was similar with the PDC group (Fig. 6c, d, e and f). The results suggested that FN-MC reduced the fatty liver and lipid droplets, repaired liver damage caused by high-fat diet, and increased the number of mitochondria and ER in the cytoplasm.

## Discussion

According to the existing study, long-term high-fat diet can lead to the increased blood lipid levels [17–19]. In our experiment, we establish hyperlipidemic rat models by feeding high fat diet to explore the therapeutic effect of FN-MC on hyperlipidemia. Our pharmacological test indicated that FN-MC was sufficient to reduce the TC, TG, LDL-c and ApoB levels and increase the HDL-c, ApoA1 and ApoA1/ApoB level in hyperlipidemic rats. Compared with HFD group, the content of MDA in the liver of FN-MC group was decreased and the activity of SOD of FN-MC group was increased. By observing the ultrastructure of liver tissue, we found that after the treatment with FN-MC, the lipid droplets were significantly reduced and the number of ER in the cytoplasm increased. These results suggested that FN-MC exerted hypolipidemic and antioxidant effects and may have a protective effect against atherosclerosis which is similar to simvastatin, the positive drug [20–22]. In addition, after 6 weeks of gavage, the weight gain of the FN-MC group was significantly lower than that of the HFD group, indicating that FN-MC has anti-obesity effects.

It is well established that long-term high blood lipids can lead to liver fibrosis; high-fat diet can induce fatty liver and liver damage. Mitochondria are organelles that provide ATP for life activities. Cell energy metabolism also relies heavily on its beta oxidation of fatty acids. It has been reported that hyperlipidemic patients have ultrastructural mitochondrial changes. Mitochondrial dysfunction can impair the homeostasis of fatty liver and induce excessive production of ROS, thereby triggering lipid peroxidation, cytokine release and cell death. [23] ER is responsible for lipid, protein synthesis, modification and transport. Studies have shown that the structural and functional abnormalities of ER are associated with hyperlipidemia. Through the observation and comparison of liver tissue sections, we confirmed that rats of HFD group had increased lipid droplets, large amounts of liver damage, reduced mitochondria and endoplasmic reticulum. After treatment with FN-MC, the fatty liver was reduced, lipid droplets were significantly reduced, liver damage caused by high-fat diet was repaired, and the number of mitochondria and ER in the cytoplasm was increased.

Changes in blood lipid levels are major risk factors for lipid metabolism disorders; elevated serum TC, TG and LDL-C levels and decreased HDL-C levels are major symptoms of hyperlipidemia [24]. Consistent with previous studies, the high-fat diet induced elevated levels of serum TC, TG, and LDL-C in the HFD group [25]. Serum LDL-C and TG levels are risk indicators for atherosclerotic cardiovascular disease. [26] Lowering serum LDL-C and TG levels may improve the risk of vascular disease and reduce the incidence of acute coronary events. [27] In our study, serum TC, TG, LDL-c levels were significantly lower in the FN-MC group compared with the HFD group, while HDL-c levels were significantly elevated, similar to the PDC group.

Apolipoprotein is a protein component that constitutes plasma lipoprotein which is to transport lipids and stabilize lipoproteins. ApoA1 is mainly derived from HDL-C, and ApoB is mainly derived from LDL-C. Therefore, decreased ApoA1 and increased ApoB are the manifestation of hyperlipidemia. ApoA1/ApoB is a risk factor for atherosclerotic cardiovascular disease [28]. The results of this study showed that FN-MC increased serum ApoAl, decreased ApoB and increased the ratio of ApoA1/ApoB. This suggested that FN-MC had regulatory effects on the apolipoprotein level of high-fat diet mice, which indicates the regulation of blood lipids.

Oxidative stress is caused by an imbalance between ROS production and the ability of those antioxidant enzymes [29]. Oxidative stress can cause damage to body cells and aggravate several diseases such as atherosclerosis and coronary heart disease [30–32]. As a typical antioxidant enzyme, SOD removes superoxide radicals by catalysis [33]. After the treatment of the FN-MC for 3 weeks, the activity of SOD was increased significantly in FN-MC group compared with HFD group. MDA is a kind of production of lipid peroxidation. The increase of MDA plays an important role in liver damage caused by hyperlipidemia. The degree of lipid peroxidation can be reflected by detecting the amount of MDA. [34] In our experiment, MDA was significantly increased in HFD group. However, following the treatment of FN-MC, MDA level decreased significantly. These results demonstrated that FN-MC can improve the activity of antioxidant enzymes in the liver of hyperlipidemic rats and reduce the content of lipid peroxidation products, so as to improve the antioxidant capacity of rats. We speculated that FN-MC protected the liver by mitigating the degree of lipid peroxidation, reducing the degree of liver cell damage and degeneration, and promoting its regeneration.

Meanwhile, it was found that the FN-MC had an effect on the weight gain of the hyperlipidemic model rats. Consistent with a previous study, the high-fat diet induced an increase in body weight [25]. The weight gain of the HFD group was significantly higher than that of the other 4 groups. The weight gain in FN-MC group (4 g/Kg) was much lower than that in HFD group, and FN-MC (2 g/Kg) also had a certain effect on decreasing weight gain. However, FN-MC had no significant effects on food intake. This indicated that FN-MC had an anti-obesity effect which might be due to the fact that the FN-MC can promote the decomposition and utilization of excess fat in the body.

## Conclusion

The results suggested that FN-MC could prevent weight gain, reduce serum levels of lipid profiles, prevent hepatic fatty deposition effectively. Meanwhile, FN-MC increased the activity of antioxidant enzymes, decreased lipid peroxidation and exerted antioxidant properties, possibly preventing the progress of hyperlipidemia. Therefore, FN-MC is a potential food additive or pharmaceutical agent to treat or prevent hyperlipidemia. Further studies of hyperlipidemia, antioxidant and anti-obesity mechanism of FN-MC are necessary.

## Abbreviations

ApoA1: Apolipoprotein A1
ApoB: Apolipoprotein B
FN-MC: *Fragarianilgerrensis* Schlecht. medicine compound
HDL-C: High-density lipoprotein
HFD: High-fat diet
LDL-C: Low-density lipoprotein
MDA: Hepatic malondialdehyde
NFD: Normal-fat diet
PDC: Positive drug control, simvastatin
SOD: Hepatic Superoxide Dismutase
TC: Total cholesterol
TG: Triglyceride

## References

[1] Jain KS, Kathiravan MK, Somani RS, Shishoo CJ (2007). The biology and chemistry of hyperlipidemia. Bioorg Med Chem.

[2] Farnier M, Davignon J (1998). Current and future treatment of hyperlipidemia: the role of statins. Am J Cardiol.

[3] Fazio S, Linton MF (2004). The role of fibrates in managing hyperlipidemia: mechanisms of action and clinical efficacy. Curr Atheroscler Rep.

[4] Zambon A, Zhao XQ, Brown BG (2014). Effects of niacin combination therapy with statin or bile acid resin on lipoproteins and cardiovascular disease. Am J Cardiol.

[5] Qiu-yu XU, Yin-hui LIU, Qi ZHANG, Bo MA, Zhen-dong YANG, Lei LIU, Di YAO, Guang-bo CUI, Jing-jing SUN, Zi-mei WU (2014). Metabolomic analysis of simvastatin and fenofibrate intervention in high-lipid diet-induced hyperlipidemia rats. Acta Pharmacol Sin.

[6] An W, Yang J (2006). Protective effects of ping-lv-mixture (PLM), a medicinal formula on arrhythmias induced by myocardial ischemia-reperfusion. J Ethnopharmacol.

[7] Wu L, Qiao H, Li Y, Li L (2007). Cardioprotective effects of the combined use of puerarin and Danshensu on acute ischemic myocardial injury in rats. Phytother Res.

[8] Kim OT, Jin ML, Lee DY, Jetter R (2017). Characterization of the Asiatic Acid Glucosyltransferase, UGT73AH1, Involved in Asiaticoside Biosynthesis in Centella asiatica (L.) Urban. Int J Mol Sci.

[9] James JT, Dubery IA (2009). Pentacyclic triterpenoids from the medicinal herb, Centella asiatica (L.) urban. Molecules.

[10] Kuang W, Zhang X, Lan Z. Flavonoids extracted from Linaria vulgaris protect against hyperlipidemia and hepatic steatosis induced by western-type diet in mice. Arch Pharm Res. 2017. 10.1007/s12272-017-0941-y. 10.1007/s12272-017-0941-y 28770537

[11] Liu C, Ma J, Sun J, Cheng C, Feng Z, Jiang H, Yang W (2017). Flavonoid-rich extract of Paulownia fortunei flowers attenuates diet-induced hyperlipidemia, hepatic steatosis and insulin resistance in obesity mice by AMPK pathway. Nutrients.

[12] Spim SRV, de Oliveira BGCC, Leite FG, Gerenutti M, Grotto D (2017). Effects of Lentinula edodes consumption on biochemical, hematologic and oxidative stress parameters in rats receiving high-fat diet. Eur J Nutr.

[13] Gao Y, Chu S, Shao Q, Zhang M, Xia C, Wang Y, Li Y, Lou Y, Huang H, Chen N (2017). Antioxidant activities of ginsenoside Rg1 against cisplatin-induced hepatinjury through Nrf2 signaling pathway in mice. Free Radic Res.

[14] Yang Q, Wang F, Rao J (2016). Effect of putrescine treatment on chilling injury, fatty acid composition and antioxidant system in kiwifruit. PLoS One.

[15] Huang S, Liu H, Meng N (2017). Hypolipidemic and antioxidant effects of Malus toringoides (Rehd.) Hughes leaves in high-fat-diet-induced Hyperlipidemic rats. J Med Food.

[16] Zhao MJ, Wang SS, Yao J (2017). Hypolipidemic effect of XH601 on hamsters of hyperlipidemia and its potential mechanism. Lipids Health Dis.

[17] Ballantyne CM, Grundy SM, Oberman A, Kreisberg RA, Havel RJ, Frost PH, Haffner SM (2000). Hyperlipidemia: diagnostic and therapeutic perspectives. J Clin Endocrinol Metab.

[18] Rachh PR, Rachh MR, Ghadiya NR, Modi DC, Modi KP, Patel NM (2010). Antihyperlipidemic activity of Gymenma sylvestre R.Br. leaf extract on rats fed with high cholesterol diet. Int J Pharmacol.

[19] Kalita H, Boruah DC, Deori M (2016). Antidiabetic and Antilipidemic effect ofMusa balbisianaRoot extract: a potent agent for glucose homeostasis in Streptozotocin-induced diabetic rat. Front Pharmacol.

[20] Sniderman AD (2004). Applying apoB to the diagnosis and therapy of the atherogenic dyslipoproteinemias: a clinical diagnostic algorithm. Curr Opin Lipidol.

[21] Brunzell JD (2005). Increased apo B in small dense LDL particles predicts premature coronary artery disease. Arterioscler Thromb Vasc Biol.

[22] Durrington P (2003). Dyslipidaemia. Lancet.

[23] Begriche K, Igoudjil A, Pessayre D (2006). Mitochondrial dysfunction in NASH: causes, consequences and possible means to prevent it. Mitochondrion.

[24] Manting L, Haihong Z, Jing L (2011). The model of rat lipid metabolism disorder induced by chronic stress accompanying high-fat-diet. Lipids Health Dis.

[25] Méndez L, Pazos M, Molinar-Toribio E (2014). Protein carbonylation associated to high-fat, high-sucrose diet and its metabolic effects. J Nutr Biochem.

[26] Deedwania PC, Pedersen TR, Demicco DA (2016). Differing predictive relationships between baseline LDL-C, systolic blood pressure, and cardiovascular outcomes. Int J Cardiol.

[27] Wada H, Dohi T, Miyauchi K (2017). Pre-procedural neutrophil-to-lymphocyte ratio and long-term cardiac outcomes after percutaneous coronary intervention for stable coronary artery disease. Atherosclerosis.

[28] Andrikoula M, Mcdowell IF (2010). The contribution of ApoB and ApoA1 measurements to cardiovascular risk assessment. Diabetes Obes Metab.

[29] Bedard K, Krause KH (2007). The NOX family of ROS-generating NADPH oxidases: physiology and pathophysiology. Physiol Rev.

[30] Firuzi O, Spadaro A, Spadaro C, Riccieri V, Petrucci R, Marrosu G, Saso L (2008). Protein oxidation markers in the serum and synovial fluid of psoriatic arthritis patients. J Clin Lab Anal.

[31] Harrison D, Griendling KK, Landmesser U, Hornig B, Drexler H (2003). Role of oxidative stress in atherosclerosis. Am J Cardiol.

[32] Bagri P, Ali M, Aeri V, Bhowmik M, Sultana S (2009). Antidiabeticeffect of Punica granatum flowers: effect on hyperlipidemia, pancreatic cells lipid peroxidation and antioxidant enzymes in experimental diabetes. Food Chem Toxicol.

[33] Arivazhagan P, Thilakavathy T, Panneerselvam C (2000). Antioxidant lipoate and tissue antioxidants in aged rats. J Nutr Biochem.

[34] Ilhan N, Halifeoglu I, Ozercan HI (2010). Tissue malondialdehyde and adenosine triphosphatase level after experimental liver ischaemia-reperfusion damage. Cell Biochem Funct.

